# Tuberculosis is always a possibility (even in the intensive care unit)

**DOI:** 10.5935/0103-507X.20160021

**Published:** 2016

**Authors:** Rodrigo Cavallazzi, Rosemeri Maurici, Julio A. Ramirez

**Affiliations:** 1 Division of Pulmonary, Critical Care and Sleep Medicine, University of Louisville - Louisville (KY), USA.; 2 Postgraduate Program in Medical Sciences, Universidade Federal de Santa Catarina - Florianópolis, SC, Brazil.; 3 Division of Infectious Diseases, University of Louisville - Louisville (KY), USA.

## INTRODUCTION

Community-acquired pneumonia (CAP) requiring hospitalization is mainly caused by *Streptococcus pneumoniae* and respiratory viruses.^(1)^ Among patients with severe CAP, including those requiring admission to an intensive care unit, additional important pathogens include *Staphylococcus aureus*, Gram-negative bacteria, and *Legionella pneumophila*.^(1,2)^

Typically, *Mycobacterium tuberculosis* is not a pathogen that is strongly considered in patients with CAP because it is usually associated with a more protracted illness course and characteristic cavitary lesions on chest imaging. However, evidence exists that *M. tuberculosis* infection can, in fact, present with clinical manifestations consistent with CAP. For instance, using the Community-Acquired Pneumonia Organization database, which is a multinational cohort of adults hospitalized with CAP, our group found that of the 6,976 patients in the database, 60 (0.86%) had infection caused by *M. tuberculosis*.^(3)^ In a study from Malaysia that included 346 patients older than 12 years of age who were hospitalized with CAP, *M. tuberculosis* was identified in 17 (4.9%) patients.^(4)^ In a study that included 103 patients with pneumonia presenting to the emergency room of a hospital in Bronx, NY, USA, 22 (21%) patients had infection caused by *M. tuberculosis*.^(5)^ The proportion of patients with tuberculosis (TB) varied considerably among these studies, which may reflect different incidences of TB in the regions where the studies were conducted.

Clinically, it may be difficult to distinguish CAP caused by *M. tuberculosis* from CAP caused by other pathogens. As an example, relying solely on the chronicity of symptoms can be misleading. Slightly more than half of the patients in a cohort with respiratory infection caused by *M. tuberculosis* had symptoms for less than a week.^(5)^ Similarly, while radiographic findings can definitely corroborate the suspicion of TB, one should not solely rely on imaging results to exclude TB. In our study, only one patient with *M. tuberculosis* infection had cavitary lesions, although in most patients, the consolidations were in the upper lobes.^(3)^ Another study found that cavitary TB tends to be localized to the upper lobes, whereas the consolidations tend to be more evenly distributed throughout the lung lobes in tuberculous pneumonia (no cavitation).^(6)^ In HIV-infected patients, consolidation or an interstitial pattern are common radiographic manifestations of TB.^(7)^

### Relevance for the intensivist

How is the above information relevant to the intensivist? Severe CAP accounts for approximately 11% of the cases of CAP requiring hospitalization.^(8)^ Thus, the intensivist in the frontline is likely to see patients with *M. tuberculosi*s infection that present as severe CAP. The recognition of patients at risk for *M. tuberculosis* infection enables early implementation of respiratory isolation, thus preventing the exposure of other people; timely diagnostic work-up of these patients, thus allowing for early diagnosis and treatment of those infected; and the avoidance of antibiotics that decrease the ability of diagnostic tests to identify *M. tuberculosis*. The failure to recognize a patient with CAP caused by *M. tuberculosis* can have serious consequences to the patient and the healthcare workers.

### Role of clinical prediction rules

Because of the difficulty in differentiating infection caused by *M. tuberculosis* from infection caused by other pathogens on clinical grounds and the variability in practice that follows when clinical judgement alone dictates management decisions, a rational approach would be to identify risk factors that are present in patients at higher risk for *M. tuberculosis* infection and then systematically apply these factors to recognize those patients. To this end, our group identified 5 factors that are independently associated with *M. tuberculosis* infection in patients with CAP as follows: (1) hemoptysis; (2) upper lobe infiltrate localization; (3) weight loss or 10% or less of ideal body weight; (4) prior history of TB or recent exposure to TB or history of positive PPD; and (5) night sweats. A risk score representing the sum of these factors led to an area under the curve of 0.89 (95%CI 0.85 - 0.93) for the diagnosis of *M. tuberculosis* infection.^(3)^ Our risk score needs to be externally and prospectively validated, but we believe that the use of a clinical prediction rule may be particularly valuable to help with the decision to place a patient in an airborne infection isolation room, a resource that is limited in most facilities.

### Diagnostic work-up

In areas with high incidence of TB, we recommend a proactive diagnostic approach based on our experience. Just as the CAP guidelines recommend that patients with severe CAP be tested for *Legionella pneumophila* via a urinary antigen test,^(9)^ we suggest that patients with CAP admitted to the hospital should routinely be tested for *M. tuberculosis* in areas with high incidence of TB. We propose that a sputum sample with smear and culture for acid-fast bacilli should be part of the routine microbiological work-up of these patients. As a matter of perspective, according to the Centers for Disease Control and Prevention a hospital with < 200 beds receiving > 3 cases of TB in a year is a medium-risk setting for transmission. A hospital with > 200 beds receiving > 6 cases a year is also considered medium risk.^(10)^

Nucleic acid amplification tests can detect *M. tuberculosis* earlier than culture methods and should be viewed as a complementary test to sputum smear and culture. In the case of a positive acid-fast bacilli smear, a positive nucleic acid amplification test increases the positive predictive value for *M. tuberculosis* to > 95% and thus allows for earlier treatment. In the case of a negative acid-fast bacilli smear, a positive nucleic acid amplification test may justify treatment depending on the preclinical probability. Alternatively, the clinician may decide to repeat the nucleic acid amplification test. Cultures should always be obtained.^(11)^

## CONCLUSION

In summary, *M. tuberculosis* is one of the pathogens that can cause community acquired pneumonia. Although classically associated with a more protracted course, *M. tuberculosis* can present in a more florid and acute fashion. The fundamental step for its recognition is the awareness by physicians that *M. tuberculosis* can even present as community acquired pneumonia with severe sepsis. In this context, we would like to bring to mind here what in Brazil has become known, by word of mouth, as Bethlem's law: tuberculosis is always a possibility.
